# Central obesity and its association with youth physical and mental health: evidence from the Australian National Health Survey

**DOI:** 10.1186/s12916-025-04538-5

**Published:** 2025-11-26

**Authors:** Setognal B. Aychiluhm, Allen G. Ross, Vivian Isaac, Subash Thapa, Kedir Y. Ahmed

**Affiliations:** 1 Rural Health Research Institute, Charles Sturt University, Orange, NSW Australia; 2 Institute of Public Health, College of Medicine and Health Sciences, University of Gondar, Gondar, Ethiopia; 3 College of Medicine, Ajman University, Ajman, United Arab Emirates; 4 School of Allied Health, Exercise & Sports Sciences, Faculty of Science & Health, Charles Sturt University, Albury, NSW Australia; 5 Translational Health Research Institute, Western Sydney University, Campbelltown Campus, Sydney, NSW Australia

**Keywords:** Central obesity, Multimorbidity, Mental health, Youth, Australia

## Abstract

**Background:**

Body fat topography, especially visceral fat accumulation in the abdominal region, is a key risk factor for cardiometabolic and mental health illnesses. This is particularly important for children and young people, as excess abdominal fat gained early in life often leads to obesity in adulthood. This study examined the prevalence and determinants of central obesity, and its association with physical and mental health morbidity among Australian youth using nationally representative data.

**Methods:**

This cross-sectional study analysed de-identified data from 3,087 youth (aged 15–24 years) in the 2017–18 Australian National Health Survey. Central obesity was defined as a waist-to-height ratio (WHtR) of ≥ 0.5. Multimorbidity was the presence of ≥ 2 long-term physical (e.g., diabetes, hypertension) or mental (e.g., anxiety, depression) disorders, while physical–mental multimorbidity required at least one of each. A Bayesian multilevel model was employed to identify factors associated with central obesity, and logistic regression was utilised to investigate the relationship between central obesity and morbidity outcomes.

**Results:**

The overall prevalence of central obesity among Australian youth (15–24 years) was 33.1% (95% CI: 31.4–34.8). Older youth (aged 18–24 years) had significantly higher odds of central obesity (AOR = 2.31; 95% CI: 1.64–3.27), as did males (AOR = 1.73; 95% CI: 1.39–2.15), those living in the most socioeconomically disadvantaged households (AOR = 2.76; 95% CI: 1.91–4.02), those residing in major cities (AOR = 1.39; 95% CI: 1.01–1.92), and individuals with depression (AOR = 1.61; 95% CI: 1.04–2.49). Additionally, our findings revealed that central obesity was significantly associated with mental disorder (AOR = 1.25; 95% CI: 1.04–1.50), overall multimorbidity (AOR = 1.27; 95% CI: 1.01–1.59), and combined physical–mental multimorbidity (AOR = 1.56; 95% CI: 1.21–2.01).

**Conclusions:**

This study highlights central obesity as a key factor associated with both physical and mental health conditions among Australian youth. A life-course perspective that addresses social determinants of health (e.g., access to safe and affordable housing, education, and healthcare) alongside individual lifestyle factors (e.g., balanced diet and regular physical activity) may help mitigate these associations.

## Background

Obesity is a major driver of a wide range of chronic conditions, including metabolic syndrome, non-alcoholic fatty liver disease, macular degeneration, certain cancers, and mental illness [1–3]. Obesity is particularly important for children and younger populations, as excess weight gained early in life often persists into adulthood, leading to the early-onset metabolic syndrome [4]. This age group undergoes rapid physiological (e.g., growth spurts, puberty-related shifts in sex hormones), behavioural (e.g., reduced physical activity and unhealthy eating patterns), and environmental changes (e.g., urbanisation with limited safe play areas, increased availability and marketing of energy-dense foods). These changes can affect fat distribution, while declining physical activity and unhealthy eating habits further contribute to weight gain [5–7].

While total body fat is important, body fat topography (the specific distribution of fat throughout the body) has emerged as a stronger predictor of cardiometabolic risk [8–10]. In 1947, Jean Vague was the first to describe this concept, classifying individuals as having ‘apple-shaped’ (central obesity) or ‘pear-shaped’ (peripheral obesity) fat patterns [11]. Since then, numerous studies have shown that visceral or abdominal fat is more closely linked to adverse metabolic and cardiovascular outcomes than overall fat mass, including insulin resistance, type 2 diabetes, dyslipidemia, hypertension, atherosclerosis, and increased risk of cardiovascular disease and non-alcoholic fatty liver disease [12–15]. Visceral fat accumulation is also associated with incident heart failure, particularly heart failure with preserved ejection fraction and adverse cardiac structure and function [16].

Although widely used, body mass index (BMI) does not distinguish between fat types or accurately reflect visceral fat levels [15]. Individuals with the same BMI and total fat mass may carry very different levels of visceral fat and face markedly different health risks [17–20]. Direct imaging methods, such as computed tomography (CT) and magnetic resonance imaging (MRI), remain the gold standard for assessing visceral fat distribution [21]. However, their cost and impracticality limit use in large-scale or low-resource studies. Consequently, simpler anthropometric measures have been developed, with the waist-to-height ratio (WHtR) emerging as a reliable, age-independent indicator of central adiposity that often outperforms BMI, waist circumference (WC), and waist-to-hip ratio (WHR) [22].

In Australia, obesity among children and adolescents has tripled in the past three decades, with over one in four now affected [23]. As of 2022, Australia ranked 10th out of 21 OECD countries for the proportion of people aged 15 and over living with overweight or obesity (64%), exceeding the OECD average of 59% [24]. Recent reports indicated that obesity now accounts for 8.3% of the total disease burden in Australia, surpassing tobacco smoking as the leading risk factor [25]. In response, the Australian government has introduced several national strategies. The National Obesity Strategy 2022–2032 outlines a comprehensive long-term plan to address the issue [26], while the Health Star Rating System (introduced in 2014) helps consumers make informed choices by providing nutritional information on food packaging [27]. The National Preventive Health Strategy 2021–2030 sets a target to reduce physical inactivity by 15% across all age groups [28].

Despite these efforts, obesity remains a persistent and complex public health challenge, particularly for young Australians. Beyond its physical and cardiometabolic impacts, obesity is closely linked to adverse mental health outcomes [29, 30]. Body image concerns, social stigma, and low self-esteem frequently affect young people with obesity, heightening the risk of depression, anxiety, and social isolation [29]. Excess abdominal fat (central obesity), which can make body shape more visibly distinct, may contribute to stigma and body dissatisfaction, thereby increasing psychological distress in youth [31]. When these psychological issues intersect with physical comorbidities (e.g., cancer, diabetes, hypertension), the cumulative burden can be profound, widening health disparities and reducing overall well-being.

To date, no published study has examined the association between central obesity and both physical and mental morbidities among Australian youth. This age group is particularly important because late adolescence and early adulthood involve rapid changes in body composition, lifestyle behaviors, and psychosocial factors that shape long-term health outcomes [32, 33]. Early prevention strategies targeting this period are therefore crucial to mitigating future disease burden and improving overall health trajectories. The present study addresses this gap by investigating the dual burden of central obesity on physical and mental health among Australian youth aged 15–24 years using nationally representative data. Building on previous evidence, including our findings that demonstrated the importance of central obesity for cardiometabolic outcomes among adults [30, 34], this study extends the scope to younger populations, highlighting the need for earlier prevention and intervention strategies.

## Methods

### Data source

This cross-sectional study analysed de-identified data from the 2017–18 Australian National Health Survey (NHS), conducted between August 2017 and December 2018. The NHS is designed to collect comprehensive health-related data across various domains, including social determinants of health (such as education, socioeconomic disadvantage, and employment), long-term health conditions (including type 2 diabetes, hypertension, heart disease, and other cardiovascular conditions (like stroke, heart attack), and health risk factors (such as smoking, alcohol consumption, poor diet and physical inactivity) within the Australian population. The NHS is a key component of the broader Intergenerational Health and Mental Health Study, funded by the Australian Government Department of Health and administered by the Australian Bureau of Statistics (ABS) [35]. This study followed the Strengthening the Reporting of Observational Studies in Epidemiology (STROBE) reporting guideline for cross-sectional studies [36, 37].

### Study setting and sampling

NHS employed a stratified random sampling design to select participants from urban, regional, and remote areas while excluding very remote areas and discrete Aboriginal and Torres Strait Islander communities. The sampling process was based on the ABS Master Sample, a comprehensive database of private dwellings across all Australian states and territories. Households were randomly selected using a multistage area sampling design. The initial sample comprised 25,109 private dwellings, which were reduced to 21,544 after accounting for sample loss (e.g., vacant, derelict, or under-construction buildings). Of these, 16,384 dwellings (households) provided fully or adequately responding data, resulting in a final person-level sample of 21,315 respondents.

Data collection was conducted through multiple modes, including online self-completion, telephone interviews, and face-to-face interviews by ABS interviewers. However, individual questionnaires were administered exclusively through face-to-face interviews [38]. Within each selected household, one adult (18 years and older) and one child (0–17 years) were randomly selected to complete individual questionnaires. For children aged 0–14 years, a parent or guardian completed the questionnaire on their behalf. Adolescents aged 15–17 years were eligible to complete the questionnaire themselves, provided they had parental or guardian consent. In cases where consent was not provided, a parent or guardian completed the questionnaire on their behalf. To reduce non-response bias, the survey incorporated follow-up strategies, including additional contact attempts, replacement sampling, and weighting adjustments, ensuring that the final sample accurately reflected the broader Australian population. In total, this study analysed a final weighted sample of 3,087 youths aged 15–24 years [38].

### Outcome variables

This study examined four interrelated health outcomes: central obesity, multimorbidity, mental disorder, and physical–mental multimorbidity.

### Central obesity

Central obesity was assessed using the waist-to-height ratio (WHtR), calculated by dividing waist circumference by height (both in centimetres). A WHtR of ≥ 0.5 defined central obesity, consistent with international guidelines [39, 40], while values < 0.5 indicated its absence. WHtR is widely considered a more reliable indicator of obesity-related health risks than body mass index (BMI), particularly in youth, as it accounts for fat distribution relative to height. While some studies reported minor variations in optimal WHtR thresholds by age, sex, or ethnicity, the ≥ 0.5 cutoff is widely accepted as a practical marker of increased cardiometabolic risk [22, 41, 42].

### Morbidity outcomes

Chronic conditions were identified by the criteria used in the 2017–18 Australian National Health Survey (NHS), following the standardised classification framework developed by the Australian Bureau of Statistics (ABS). Respondents were asked whether they had ever been told by a doctor or nurse that they had a particular condition, whether the condition was still current, and whether it had lasted or was expected to last six months or longer. A condition was classified as chronic if it was both current and of long-term nature, that is, it had persisted or was expected to persist for at least six months [43]. This self-reported, diagnosis-based approach was applied consistently to define all chronic health outcomes assessed in this study, including physical and mental conditions. Using this framework, three morbidity-related outcomes were defined:

- Multimorbidity was defined as the coexistence of two or more chronic conditions (coded as 1), with fewer than two conditions classified as no multimorbidity (coded as 0) [44, 45]. The classification encompassed a comprehensive range of chronic diseases, including but not limited to: malignant neoplasms (e.g., colorectal cancer); cardiovascular diseases (e.g., myocardial infarction, heart failure, stroke); chronic respiratory conditions (e.g., asthma, chronic bronchitis); endocrine disorders (e.g., diabetes mellitus); gastrointestinal disorders (e.g., peptic ulcer disease); musculoskeletal disorders (e.g., osteoarthritis, rheumatoid arthritis); neurological conditions (e.g., epilepsy, multiple sclerosis); sensory impairments (e.g., hearing loss, cataracts); and mental and behavioural disorders(e.g., depression, anxiety, schizophrenia).
- Mental disorder was coded as present (1) if the respondent reported a professionally diagnosed mental or behavioural disorder and coded as absent (0) otherwise. These disorders included mood disorders (e.g., depression, bipolar disorder), anxiety disorders (e.g., generalised anxiety disorder, panic disorder), schizophrenia spectrum and other psychotic disorders, substance use disorders, intellectual disability, developmental disorders (e.g., autism spectrum disorders), and organic mental disorders (e.g., dementia).
- Physical-mental multimorbidity refers to the presence of at least one physical condition alongside a mental disorder, with 1 indicating both conditions are present, and 0 indicating either no conditions or only one of the conditions is present [46].

### Explanatory variables

Explanatory variables in this study were broadly categorised into sociodemographic (age, sex, education, income, marital status), lifestyle (physical inactivity, disability, smoking, vegetable, fruit and alcohol consumption), psychosocial (anxiety and depression), and geographic factors (remoteness, socioeconomic disadvantage). Details are provided in Table 1.

**Table 1:** Definitions of Study variables

Variables	Definition
Sociodemographic factors	
Age	1 = 15–17 years, 2 = 18–24 years
Sex	1 = Males, 2 = Females
Highest Educational Attainment	1 = Did not complete Year 12, 2 = Completed Year 12 or above
Personal Income	The 2021 census median personal income (AUD $805 per week) was used to dichotomize as 1 = Average or above, 2 = Below average [47]
Marital Status	1 = Married, 2 = Not married
Socioeconomic Disadvantage	Defined based on the Index of Relative Socio-Economic Disadvantages (IRSD). Quantile 1 = Most disadvantaged, Quantile 5 = Least disadvantaged
Main language spoken at home	1 = English, 2 = Other languages
Lifestyle risk factors	
Physical activity	Physical activity is defined based on the Australian Department of Health guidelines, 2014 [48]. It was classified as ‘1’ = Physically inactive for individuals who did not meet the 2014 physical activity guidelines, and ‘2’ = Physically active for those who met the guidelines
Fruit consumption	Fruit consumption was defined according to the National Health and Medical Research Council (NHMRC) 2013 guidelines [49]. Participants were categorized as ‘1’ = ‘Adequate/meet recommended fruit consumption’ (consuming 2 servings of fresh fruit, equivalent to 150 g, per day) or ‘2’ = ‘Inadequate/not met recommended fruit consumption’ (consuming less than the recommended 2 servings per day)
Vegetable consumption	Vegetable consumptions were defined according to the NHMRC 2013 guidelines [49]. Participants were categorized as ‘1’ = ‘Adequate/met recommended vegetable consumption’ (consuming 5 servings of vegetables per day) or ‘2’ = ‘Inadequate/not met recommended vegetable consumption’ (consuming less than the recommended 5 servings per day)
Sugar/sweetened drink consumption	Sugar/sweetened drink consumption can be categorized based on daily intake of sugary and other sugar-sweetened beverages. It is grouped as: ‘1’ = daily consumption of sugary drinks (consuming sugary beverages or other sugar-sweetened drinks daily) or ‘2’ = non-daily consumption (not consuming sugary beverages or other sugar-sweetened drinks daily)
Daily smoking	Daily smoking was categorized based on the frequency of cigarette use, with two groups: ‘1’ = ‘daily smoking’ (smoking cigarettes or using tobacco every day or most days of the week) or ‘2’ = ‘non-daily smoking’ (smoking cigarettes on some days, but not every day)
Excessive alcohol consumption	Excessive alcohol consumption is defined according to the NHMRC, 2020 [50, 51]. Grouped as ‘1’ = ‘excessive drinking’ (an individual regularly drinking more than 10 standard drinks per week (over 21 units/week) or more than 4 standard drinks on any given day] or ‘2’ = ‘non-excessive drinking’ (an individual regularly drinking no more than 10 standard drinks per week, and no more than 4 standard drinks on any one day)
Disability status	1 = Disable, 2 = Not disable
Psychosocial factors	
Depression	Depression was assessed based on patients' self-reported diagnosis, determined by whether they had been diagnosed with depression or depressive disorder by a healthcare professional. The condition must have lasted for at least six months or been expected to persist for six months or more [29, 52]. Depression was categorized as ‘1’ = ‘Yes’ and ‘2’ = ‘No’
Anxiety	Anxiety was assessed based on patients' self-reported diagnosis, determined by whether they had been diagnosed with anxiety or anxiety disorder by a healthcare professional. The condition must have lasted for at least six months or been expected to persist for six months or more [29, 53]. Anxiety was grouped as ‘1’ = ‘Yes’ and ‘2’ = ‘No’
Geographic Factor	
Remoteness	‘1’ = ‘Urban/major cities’, ‘2’ = ‘inner regional areas’, ‘3’ = ‘outer regional’, and ‘4’ = ‘remote’ (Based on Australian Statistical Geography Standard (ASGS) [35])

### Data management

All data used in this study were obtained through the Australian Bureau of Statistics (ABS) DataLab (https://new.datalab.abs.gov.au/). The processes of data cleaning, coding, and extraction were performed using STATA MP software (version 18, StataCorp, College Station, TX, USA). After cleaning, the dataset was imported into R-studio (version 4.4.1) for further statistical analysis. Initial descriptive analyses summarised study characteristics using weighted frequencies and percentages, with all calculations adjusted for the personal weight variable.

### Statistical modeling

To investigate the associations between explanatory variables and central obesity, we employed a Bayesian multilevel logistic regression model. Unlike classical logistic regression, which treats parameters as fixed and relies on maximum likelihood estimation (MLE), the Bayesian approach treats model parameters as random variables [54]. This framework allows for the incorporation of prior knowledge and provides a robust mechanism for quantifying uncertainty in parameter estimates, facilitating a more flexible and nuanced analysis [54, 55].

Bayesian methods estimate parameters through their full posterior distributions, offering advantages for complex hierarchical models [55]. Given the multistage sampling design of the NHS and potential clustering within geographic and demographic strata, we employed a Bayesian multilevel logistic regression model with random effects. This approach enhances the precision and credibility of estimated associations, enabling results to be reported as 95% credible intervals.

The Bayesian regression analysis was performed using the brms package in R [56], which utilizes Hamiltonian Monte Carlo (HMC) and the No-U-Turn Sampler (NUTS) algorithm, implemented in Stan, for efficient sampling. Regression coefficients were assigned weakly informative Normal (0, 10) priors (variance 100; SD = 10), minimising prior influence. The model was estimated using 3 independent chains, each containing 10,000 iterations, with 2,500 warmup iterations per chain. This resulted in a total of 22,500 post-warmup draws, providing a robust sample for reliable and accurate parameter estimates. This configuration was optimized to ensure convergence and minimize sampling bias.

Convergence of the Markov chains was evaluated using multiple diagnostic criteria to ensure the reliability of posterior estimates. These included R̂ (Rhat) values close to 1.00, indicating convergence across chains, and effective sample sizes (ESS) greater than 1,000 for both the bulk (Bulk_ESS) and tail (Tail_ESS) of the posterior distributions. Visual diagnostics, including trace plots and posterior density plots, were also examined for evidence of adequate mixing and stability. These plots demonstrated well-mixed chains and smooth posterior distributions, confirming satisfactory convergence (Supplementary File 1).

To assess robustness to prior specification, a sensitivity analysis was performed by refitting the model with alternative weakly informative priors: Normal (0, 10) and Normal (0, 2.5). Posterior means and 95% credible intervals for key predictors were consistent across these specifications [57].

A binary logistic regression model was used to assess the association between central obesity and the co-occurrence of physical and mental health conditions. This approach was chosen due to the absence of a clustering effect for these outcomes, making the simpler logistic regression more efficient and interpretable. Multicollinearity among predictor variables was assessed using the variance inflation factor (VIF), with all values below 5, indicating that multicollinearity was not a concern. The association was quantified using adjusted odds ratios (AORs) with corresponding 95% confidence intervals (CIs). Simultaneously, a Bayesian approach estimated the associations between central obesity and each significant explanatory variable, with results expressed as adjusted odds ratios (AORs) and 95% credible intervals (CrIs), providing a direct probabilistic interpretation of parameter uncertainty.

## Results

### Description of study participants

A total weighted sample of 3,087 youth aged 15–24 years in Australia was included in the study. The median age of participants was 20 years (Interquartile Range(IQR): 17–22), with 48.9% identifying as female. Of the participants, 17.5% were from the most disadvantaged quintile (quintile 1), while 21.2% were from the least disadvantaged quintile (quintile 5). Most participants (76.6%) resided in major cities, with 7.1% living in outer regional and remote areas. A small proportion (4.8%) of individuals met the daily vegetable consumption requirement, while nearly half (49.6%) met the recommended fruit consumption. Approximately 13.0% of participants reported consuming sugary or sweet drinks daily. Regarding psychological factors, 10.2% of participants reported experiencing anxiety, and 7.8% reported depression (Table 2).

**Table 2:** Description of the study participants among youth aged 15–24 years in Australia (*N* = 3,087)

Variable	Weighted n (%)
Central obesity	
No	2,066 (66.9)
Yes	1,021 (33.1)
Age group	
15–17 years	847 (27.4)
18–24 years	2,240 (72.6)
Sex	
Male	1,577 (51.1)
Female	1,510 (48.9)
Educational level	
Year 12 and above	1,961 (63.5)
Below year 12	1,126 (36.5)
Marital status	
Married	86 (2.8)
Not married	3,001 (97.2)
Household income	
Average or more	928 (30.1)
Below average	2,159 (69.9)
Language	
Other languages	355 (11.5)
English	2,732 (88.5)
Physical activity	
Active	473 (15.3)
Inactive	2,614 (84.7)
Disability status	
Has no disability	2,464 (79.8)
Has disability	623 (20.2)
Smoking status	
Not smoker	2,448 (79.3)
Smoker	639 (20.7)
Fruit consumption	
Met recommendations	1,530 (49.6)
Did not meet recommendations	1,557 (50.4)
Vegetable consumption	
Met recommendations	150 (4.8)
Did not meet recommendations	2,937 (95.2)
Sweet drink/consumption	
Non-daily consumers	2,683 (86.9)
Daily consumers	404 (13.1)
Alcohol drinking	
No	2,931 (95.0)
Yes	156 (5.0)
Depression	
No	2,845 (92.2)
Yes	242 (7.8)
Anxiety	
No	2,772 (89.8)
Yes	315 (10.2)
IRSD	
Quintile 5	653 (21.2)
Quintile 1	541 (17.5)
Quintile 2	614 (19.9)
Quintile 3	638 (20.7)
Quintile 4	641 (20.8)
Remoteness	
Major cities	2,364 (76.6)
Inner regional	504 (16.3)
Outer regional and remote	219 (7.1)

IRSD, index of relative socioeconomic disadvantage. Quintile 1 denotes high disadvantage and Quintile 5 denotes low disadvantage. For remoteness, outer regional and remote categories have been combined due to the small sample size. Physical activity was classified based on the Australian Department of Health (2014) guidelines as physically inactive (did not meet the guidelines) or physically active (met the guidelines). Fruit and vegetable consumption were defined according to NHMRC (2013) guidelines. Fruit intake was categorised as adequate (≥ 2 servings/day, 150 g) or inadequate (< 2 servings/day), and vegetable intake was categorised as adequate (≥ 5 servings/day) or inadequate (< 5 servings/day). Sugar-sweetened drink consumption was classified as daily (consumed every day) or non-daily (not consumed every day)

### The prevalence of central obesity

The overall prevalence of central obesity among youth aged 15–24 years in Australia was 33.1% (CI: 31.4%, 34.8%). The prevalence was higher among those aged 18–24 years (37.5%) compared to those aged 15–17 years (21.4%). Males had a higher prevalence than females (38.5% vs 27.4%). Youth with disabilities exhibited a higher prevalence of central obesity compared to their non-disabled counterparts (37.8% vs 31.9%). The highest prevalence of central obesity was observed in the most disadvantaged areas (quintile 1), at 40.8%, while the lowest prevalence was in the wealthiest areas (quintile 5), at 23.2%.

Australian youths who did not meet the recommended intake of fruit (35.7%) and vegetables (33.4%) had a higher prevalence of central obesity compared to those who met the recommended levels. Similarly, youths who consumed sugar-sweetened beverages had a higher prevalence of central obesity (38.7%) compared to those who did not consume sugar-sweetened beverages. Youths diagnosed with depression (46.2%) and anxiety (42.2%) had a higher prevalence of central obesity compared to those without these conditions. The prevalence of central obesity was higher in major cities (33.7%) compared to outer regional and remote areas (26.6%) [Table 3].

**Table 3:** Prevalence of central obesity among youth (15–24 years) in Australia (*N* = 3,087)

Variable	Weighted	
	No n (%)	Yes n (%)
Age group		
15–17 years	666 (78.6)	181 (21.4)
18–24 years	1,400 (62.5)	840 (37.5)
Sex		
Male	970 (61.5)	607 (38.5)
Female	1,096 (72.6)	414 (27.4)
Educational level		
Year 12 and above	1,263 (64.4)	698 (35.6)
Below year 12	803 (71.3)	323 (28.7)
Marital status		
Married	43 (49.4)	43 (50.6)
Not married	2,023 (67.4)	978 (32.6)
Household income		
Average or more	674 (72.6)	254 (27.4)
Below average	1,392 (64.5)	767 (35.5)
Language		
Other languages	227 (63.9)	128 (36.1)
English	1,839 (67.3)	893 (32.7)
Physical activity		
Active	319 (67.4)	154 (32.6)
Inactive	1,747 (66.8)	867 (33.2)
Disability status		
Has no disability	1,679 (68.1)	785 (31.9)
Has disability	387 (62.2)	236 (37.8)
Smoking status		
Not smoker	1,699 (69.4)	748 (30.6)
Smoker	367 (57.3)	273 (42.7)
Fruit consumption		
Met recommended	1,064 (69.6)	466 (30.4)
Not met recommended	1,002 (64.3)	555 (35.7)
Vegetable consumption		
Met recommended	111 (74.1)	39 (25.9)
Not met recommended	1,955 (66.6)	982 (33.4)
Sweet drink/consumption		
Not drink	1,819 (67.8)	865 (32.2)
Drink	247 (61.3)	156 (38.7)
Alcohol drinking		
No	1,961 (66.9)	970 (33.1)
Yes	105 (67.1)	51 (32.9)
Depression		
No	1,936 (68.0)	910 (32.0)
Yes	130 (53.8)	111 (46.2)
Anxiety		
No	1,884 (67.9)	888 (32.1)
Yes	182 (57.8)	133 (42.2)
IRSD		
Quintile 5	501 (76.8)	152 (23.2)
Quintile 1	320 (59.2)	221 (40.8)
Quintile 2	386 (62.8)	228 (37.2)
Quintile 3	420 (65.8)	218 (34.2)
Quintile 4	439 (68.5)	202 (31.5)
Remoteness		
Major cities	1,567 (66.3)	797 (33.7)
Inner regional	338 (67.1)	166 (32.9)
Outer regional and remote	161 (73.4)	58 (26.6)

IRSD, index of relative socioeconomic disadvantage. Quintile 1 denotes high disadvantage and Quintile 5 denotes low disadvantage. For remoteness, outer regional and remote categories have been combined due to the small sample size

### The prevalence of physical-mental morbidity

The overall prevalence of multimorbidity, mental disorder, and physical mental multimorbidity among Australian youths was 15.3%, 25.4% and 11.5%, respectively. The prevalence of multimorbidity was higher among those with central obesity compared to those who did not have central obesity (17.9% vs 14.0%). Similarly, mental health conditions were more commonly reported by individuals with central obesity (28.1%) than those without central obesity (24.1%). The prevalence of physical-mental multimorbidity, defined as the coexistence of chronic physical conditions and mental disorders, was also higher among individuals with central obesity compared to their counterparts (14.3% vs 10.1%) [Table 4].

**Table 4:** Weighted Distribution of Multimorbidity and Mental Health Conditions by Obesity Status Among Youth Aged 15–24 Years in Australia (Total = 3087)

Condition	Overall (n, %)	Central Obesity No (n, %)	Central Obesity Yes (n, %)	General Obesity No (n, %)	General Obesity Yes (n, %)
Multimorbidity					
Yes	471 (15.3%)	288 (14.0%)	183 (17.9%)	384 (14.5%)	87 (20.3%)
No	2,616 (84.7%)	1,777 (86.0%)	839 (82.1%)	2,274 (85.5%)	342 (79.7%)
Mental disorder					
Yes	785 (25.4%)	498 (24.1%)	287 (28.1%)	638 (24.0%)	147 (34.2%)
No	2,302 (74.6%)	1,568 (75.9%)	734 (71.9%)	2,020 (76.0%)	282 (65.8%)
Phys–Mental Multimorbidity					
Yes	354 (11.5%)	208 (10.1%)	146 (14.3%)	278 (10.5%)	76 (17.7%)
No	2,733 (88.5%)	1,858 (89.9%)	875 (85.7%)	2,380 (89.5%)	353 (82.3%)

### Factors associated with central obesity

Our findings showed that older youths aged 18–24 years had significantly higher odds of central obesity compared to those aged 15–17 years (AOR = 2.31; 95% CI: 1 64, 3.27), and males were more likely to have central obesity compared to females (AOR = 1.73; 95% CI: 1.39, 2.15). Youth who resided in the most socioeconomically disadvantaged areas (quintile 1) had significantly higher odds of central obesity compared to those in the wealthiest areas (quintile 5) (AOR = 2.76; 95% CI: 1.91–4.02). A dose–response relationship was observed across socioeconomic quintiles, with increased odds also seen in quintile 2 (AOR = 2.02; 95% CI: 1.40–2.91), quintile 3 (AOR = 1.73; 95% CI: 1.20–2.48), and quintile 4 (AOR = 1.59; 95% CI: 1.11–2.29) [Table 5).

**Table 5:** Bayesian multilevel multivariable logistic regression model for factors associated with central obesity among youth in Australia (Total = 3087)

Variable	AOR (95%CI)	SE
Age group		
15–17 years	1	
18–24 years	2.31 (1.64, 3.27) *	0.174
Sex		
Female	1	
Male	1.73 (1.39, 2.15) *	0.110
Educational level		
Year 12 and above	1	
Below year 12	1.10(0.82, 1.49)	0.150
Household income		
Average or more	1	
Below average	1.05 (0.81, 1.35)	0.130
Language		
Other languages	1	
English	1.22 (0.84, 1.77)	0.191
Physical activity		
Active	1	
Inactive	1.26 (0.91, 1.77)	0.169
Disability status		
Has no disability	1	
Has disability	0.98 (0.75, 1.28)	0.136
Smoking status		
Not-smoker	1	
Smoker	1.13 (0.85, 1.49)	0.141
Depression		
No	1	
Yes	1.61 (1.04, 2.49) *	0.222
Anxiety		
No	1	
Yes	1.24 (0.82, 1.85)	0.207
Fruit consumption		
Met recommended	1	
Not met recommended	1.12 (0.90, 1.39)	0.110
Vegetable consumption		
Met recommended	1	
Not met recommended	1.16 (0.72, 1.91)	0.250
Sweet dink/consumption		
Not drink	1	
Drink	0.82 (0.60, 1.10)	0.155
IRSD		
Quintile 5	1	
Quintile 1	2.76 (1.91, 4.02) *	0.190
Quintile 2	2.02 (1.40, 2.91) *	0.186
Quintile 3	1.73 (1.20, 2.48) *	0.183
Quintile 4	1.59 (1.11, 2.29) *	0.184
Remoteness		
Outer regional and remote	1	
Major cities	1.39(1.01, 1.92) *	0.165
Inner regional	1.00 (0.68, 1.47)	0.195

^*^Statistically significant variables at 95% CI, AOR = adjusted odds ratio, CI = credible interval, SE = standard error, RSD, index of relative socioeconomic disadvantage. Quintile 1 denotes high disadvantage and Quintile 5 denotes low disadvantaged. For remoteness, outer regional and remote categories have been combined due to the small sample size

Living in major cities was associated with significantly higher odds of central obesity compared to residing in outer regional and remote areas (AOR = 1.39; 95% CI: 1.01–1.92). In addition, those with depression had significantly higher odds of central obesity compared to those without depression (AOR = 1.61; 95% CI: 1.04–2.49) [Table 5].

### Associations between obesity, physical and mental health morbidity

**Fig. 1:**
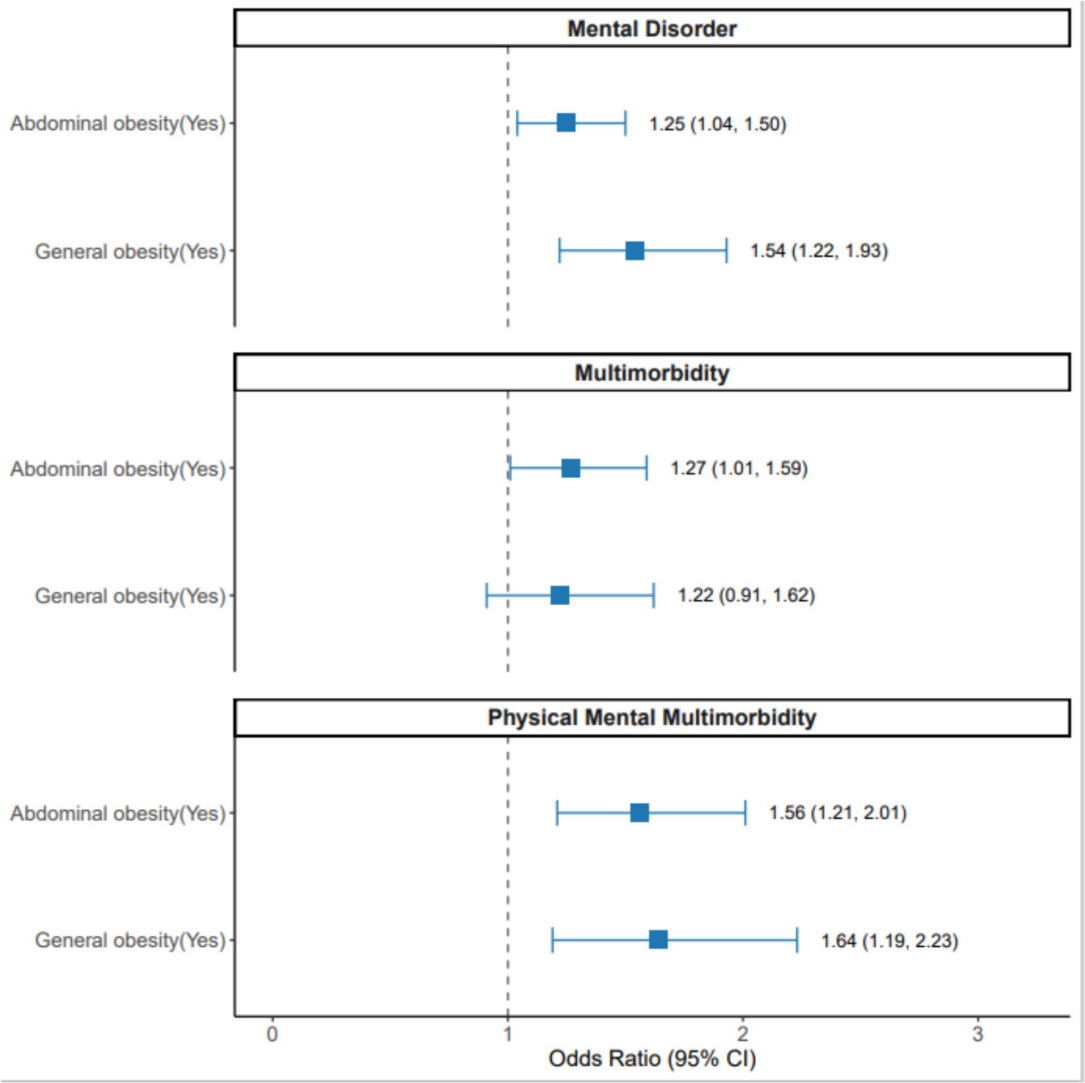
Associations between of abdominal and general obesity with physical and mental morbidity among youth in Australia (weighted) (Total = 3087). Note: No central obesity was the reference for central obesity (Yes) and No general obesity was the reference for general obesity (Yes)

This study found that both central obesity (AOR = 1.25; 95% CI: 1.04–1.50) and general obesity (AOR = 1.54; 95% CI: 1.22–1.93) were positively associated with mental disorder among youth. Additionally, both central obesity (AOR = 1.56; 95% CI: 1.21–2.01) and general obesity (AOR = 1.64; 95% CI: 1.19–2.23) were significantly associated with physical-mental multimorbidity. Central obesity was also significantly associated with overall multimorbidity (AOR = 1.27; 95% CI: 1.01–1.59), while general obesity did not show a statistically significant association with overall multimorbidity (AOR = 1.22; 95% CI: 0.91–1.62) [Fig. 1].

## Discussion

This study examined the prevalence and determinants of central obesity and its association with physical and mental morbidity among Australian youth aged 15–24 years. The findings provide nationally representative evidence on central obesity in a population group that has been largely overlooked in obesity research [58, 59]. This age group represents a critical period during which rapid changes in body composition, lifestyle behaviours, and psychosocial factors shape long-term health trajectories [58, 59]. In distinction from prior studies focusing on BMI-defined general obesity [60, 61] or on school-aged children [62, 63], we employed WHtR, a more sensitive and clinically meaningful indicator of metabolic and cardiovascular risk. By concurrently assessing the association between central obesity and both physical and mental health outcomes, our findings reveal the intertwined burden of central obesity and multimorbidity in young people.

Our findings revealed that one in three youths aged 15–24 years had central obesity, with a higher prevalence observed among males. Older youth (aged 18–24), males, individuals from socioeconomically disadvantaged households, and those living in major cities or with depression were more likely to have central obesity. Furthermore, central obesity was significantly associated with mental disorders, multimorbidity, and combined physical–mental multimorbidity.

These results are consistent with both national and global evidence, which reports a substantial burden of central obesity among young adults, particularly among males and older adolescents [61, 64]. In Australia, recent data highlight a high prevalence of central obesity, particularly among males, young adults, and individuals from socioeconomically disadvantaged households [30, 65, 66]. Moreover, central obesity is strongly linked with adverse health outcomes: several studies demonstrate associations with mental disorders such as depression and anxiety [67] as well as chronic physical conditions, including hypertension, type 2 diabetes, and cardiovascular diseases [30, 68, 69].

Observed sex differences may reflect biological and developmental patterns that emerge in late adolescence, during which males tend to accumulate more visceral fat due to hormonal influences and changes in body composition [70].The association between central obesity and youths residing in major cities is potentially reflecting lifestyle patterns, including greater exposure to ultra-processed foods, engage in more sedentary behaviours, and spend more time in screen-based activities, with fewer opportunities for outdoor physical activity [71, 72]. Similarly, the link between central obesity and socioeconomic disadvantage suggests that young people living in low-income areas, where fast-food outlet density tends to be higher, may be at greater risk of developing central obesity [73].

The strong link between central obesity and mental disorders highlights a bidirectional relationship between physical and psychological health. Obesity can negatively affect mental wellbeing through mechanisms such as social stigma, low self-esteem, emotional dysregulation, and reduced physical activity [74]. In turn, mental disorders, particularly depression, can contribute to weight gain through emotional eating and reduced motivation for physical activity [75]. This self-reinforcing cycle exacerbates both conditions, leading to persistent morbidity and diminished quality of life [74, 75].

Mental disorders remain a major public health issue for Australian youth, representing the leading cause of disease burden in this population [76, 77]. According to the Australian Burden of Disease Study 2024, mental health and substance use disorders together contribute to roughly 15% of the total disease burden among young Australians [25]. These challenges are driven by a complex interplay of socio-environmental factors and insufficient access to age-appropriate public health services [78]. The rising prevalence of obesity, particularly central obesity, further compounds this problem. Central obesity shares common psychosocial risk factors with mental disorders and interacts bi-directionally with them [79].

Central obesity was also independently associated with multimorbidity and combined physical–mental health conditions. Visceral adiposity contributes to systemic inflammation, insulin resistance, and neurohormonal changes [80], and emerging evidence suggests neuroinflammatory pathways may link it to common mental disorders [81]. Beyond biological mechanisms, social determinants, such as socioeconomic disadvantage, limited access to healthy foods, and insufficient opportunities for physical activity, further contributed to the risk.

### Implications of the study findings

Early-life central obesity is associated with adverse physical and psychosocial outcomes that can persist into adulthood and across generations [82–87], highlighting the importance of prevention and early intervention. Adolescents with obesity are much more likely to remain obese as adults, thereby increasing the burden of chronic diseases at the population level and associated future healthcare costs [86, 88].

The disproportionate impact of central obesity on disadvantaged youth underscores the urgent need for multicomponent public health strategies that address structural and socioeconomic barriers [89]. These multicomponent interventions should prioritise early prevention and promote healthy lifestyles through school- and community-based programs [90]. Public health policies are required to improve access to nutritious foods, reduce the consumption of sugar-sweetened beverages and ultra-processed foods, and create environments that encourage regular physical activity.

Finally, the bidirectional relationship between central obesity and mental disorders calls for the integration of mental health services within obesity prevention and management initiatives. Such strategies should particularly consider individuals with central obesity who may have a normal BMI, as this group often remains undetected and underserved in current public health programs.

### Strengths and limitations of the study

This study draws on data from the Australian National Health Survey, a robust, nationally representative resource for understanding health outcomes at the population level. The use of waist-to-height ratio (WHtR), a validated and superior measure of abdominal obesity, enhances the clinical relevance of our findings. By examining both physical and mental health outcomes, the study provides a comprehensive view of multimorbidity, supporting integrated, person-centred prevention strategies. Advanced Bayesian multilevel modeling with HMC/NUTS ensures robust parameter estimation and transparent uncertainty quantification. Applying survey weights and adjustments for complex sampling design further strengthens the validity and generalizability of the results.

Despite these strengths, the study has several limitations. The cross-sectional nature of the study design limits the ability to infer causality between explanatory variables and outcomes. While WHtR is a reliable indicator of abdominal obesity, it does not account for variations in body fat distribution, which may affect its accuracy in some populations. Moreover, we applied the widely accepted WHtR ≥ 0.5 threshold, although optimal cut-offs may vary by age, sex, and population subgroup, which may influence generalizability. The reliance on self-reported data introduces potential biases, including recall bias, misunderstanding of survey questions, and social desirability bias. Finally, although sociodemographic factors were controlled for, residual confounding from unmeasured variables, such as genetic predispositions, environmental factors, and cultural or linguistic background, may still influence the results. Future studies should use longitudinal and qualitative approaches, including biomarker analyses, to explore the experiences of youth with central obesity, stigma, and mental disorders, providing context for the observed associations and informing evidence-based public health policies.

## Conclusion

Our study shows that one in three Australian youth has central obesity, with higher prevalence among males and those from socioeconomically disadvantaged backgrounds. Central obesity is strongly associated with poor health, including mental disorders and combined physical–mental multimorbidity. Given that late adolescence and early adulthood are critical periods for the development of chronic conditions, early prevention is important. Global evidence highlights that lifestyle interventions can effectively reduce central obesity. Addressing shared risk factors through life-course and equity-focused strategies may provide an effective approach to preventing both chronic physical and mental health conditions in youth.

## Data Availability

The data used in this study are available from the ABS. Due to ABS privacy and confidentiality policies, the microdata cannot be publicly shared. Access requires a formal application and approval through the ABS DataLab, and all outputs generated within the DataLab are subject to ABS review prior to release.

## Abbreviations

ABS: Australian Bureau of Statistics
AOR: Adjusted Odds Ratio
CI: Confidence Interval
CrI: Credible Interval
ESS: Effective Sample Size
HMC: Hamiltonian Monte Carlo
IQR: Interquartile Range
MLE: Maximum Likelihood Estimation
NHS: National Health Survey
NUTS: No-U-Turn Sampler
R̂: Gelman–Rubin convergence statistic
STROBE: Strengthening the Reporting of Observational Studies in Epidemiology
VIF: Variance Inflation Factor
WC: Waist Circumference
WHtR: Waist-to-Height Ratio
WHO: World Health Organization
